# A high diversity of non-target site resistance mechanisms to acetolactate-synthase (ALS) inhibiting herbicides has evolved within and among field populations of common ragweed (*Ambrosia* *artemisiifolia* L.)

**DOI:** 10.1186/s12870-023-04524-0

**Published:** 2023-10-24

**Authors:** Ingvild Loubet, Lucie Meyer, Séverine Michel, Fanny Pernin, Sébastien Carrère, Benoit Barrès, Valérie Le Corre, Christophe Délye

**Affiliations:** 1 INRAE, Agroécologie, Dijon, France; 2 Université de Lyon, Anses, INRAE, USC CASPER, Lyon, France; 3 INRAE, LIPM-Bioinfo, Toulouse, France

**Keywords:** *Ambrosia artemisiifolia*, Acetolactate-synthase (ALS) inhibitor, Herbicide, Non-target-site resistance, RNA sequencing, Transcriptomics, Resistance detection, Adaptive evolution

## Abstract

**Background:**

Non-target site resistance (NTSR) to herbicides is a polygenic trait that threatens the chemical control of agricultural weeds. NTSR involves differential regulation of plant secondary metabolism pathways, but its precise genetic determinisms remain fairly unclear. Full-transcriptome sequencing had previously been implemented to identify NTSR genes. However, this approach had generally been applied to a single weed population, limiting our insight into the diversity of NTSR mechanisms. Here, we sought to explore the diversity of NTSR mechanisms in common ragweed (*Ambrosia artemisiifolia* L.) by investigating six field populations from different French regions where NTSR to acetolactate-synthase-inhibiting herbicides had evolved.

**Results:**

A de novo transcriptome assembly (51,242 contigs, 80.2% completeness) was generated as a reference to seek genes differentially expressed between sensitive and resistant plants from the six populations. Overall, 4,609 constitutively differentially expressed genes were identified, of which none were common to all populations, and only 197 were shared by several populations. Similarly, population-specific transcriptomic response was observed when investigating early herbicide response. Gene ontology enrichment analysis highlighted the involvement of stress response and regulatory pathways, before and after treatment. The expression of 121 candidate constitutive NTSR genes including CYP71, CYP72, CYP94, oxidoreductase, ABC transporters, gluco and glycosyltransferases was measured in 220 phenotyped plants. Differential expression was validated in at least one ragweed population for 28 candidate genes. We investigated whether expression patterns at some combinations of candidate genes could predict phenotype. Within populations, prediction accuracy decreased when applied to an additional, independent plant sampling. Overall, a wide variety of genes linked to NTSR was identified within and among ragweed populations, of which only a subset was captured in our experiments.

**Conclusion:**

Our results highlight the complexity and the diversity of NTSR mechanisms that can evolve in a weed species in response to herbicide selective pressure. They strongly point to a non-redundant, population-specific evolution of NTSR to ALS inhibitors in ragweed. It also alerts on the potential of common ragweed for rapid adaptation to drastic environmental or human-driven selective pressures.

**Supplementary Information:**

The online version contains supplementary material available at 10.1186/s12870-023-04524-0.

## Introduction

Common ragweed (*Ambrosia artemisiifolia* L.) is an invasive plant species native from North America [1] that has spread in Europe through multiple introductions [2]. Widely dispersed by human activities [3, 4], ragweed preferentially colonises disturbed environments [3], including agricultural fields. Because of its highly allergenic and allergy-inducing pollen, ragweed spread and proliferation are major public health concerns [5]. Furthermore, ragweed is a serious agricultural weed that can cause heavy yield losses in summer crops such as maize, soybean or sunflower [6, 7]. Thus, ragweed control in cropped areas is of primary importance for both public health and farming competitiveness. In arable fields, ragweed is mainly controlled by herbicides. The most effective crop-selective herbicides against ragweed target acetolactate-synthase (ALS), a key enzyme in the biosynthesis of branched-chain amino acids [8]. In France, the ALS inhibitors imazamox, an imidazolinone, and tribenuron, a sulfonylurea, have broadly been applied since 2010 to curb ragweed infestations, especially in fields cultivated with herbicide-tolerant sunflower varieties. However, a lack of diversified agronomic practices set a high risk for resistance evolution in weeds [9]. In France, the first cases of ragweed resistance to ALS inhibitors were observed in 2013 [10]. The genetic determinants of herbicide resistance can be mutant alleles of the herbicides target gene (target-site-based resistance, TSR), and/or alleles of genes involved in plant secondary metabolism (non-target-site-based resistance, NTSR) [11]. Both types of resistance mechanisms have evolved in ragweed in France: while a diversity of TSR alleles has been identified in a large sample of ragweed field populations [12], two recent studies have shown that NTSR was by far the most widespread and frequent type of resistance [10, 12]. In addition, both studies suggested that a diversity of NTSR mechanisms had evolved among the surveyed populations. This prompted us to investigate the genetic bases of NTSR to ALS inhibitors in ragweed.

It has been proposed that NTSR is largely due to exacerbated herbicide detoxification and/or compartmentation, likely controlled by alleles at gene families involved in plant secondary metabolism that carry structural and/or regulatory mutations [11, 13]. Whole-transcriptome scanning approaches comparing resistant and sensitive plants are thus methods of choice to identify NTSR genetic bases [13]. Quite a few transcriptomic studies have been conducted to investigate the genetic determinisms of NTSR in various weed species (Table S1). Most NTSR genetic determinants identified so far belong to xenobiotic detoxification or defence pathways, which is consistent with exacerbated herbicide metabolism playing a major role in NTSR (*e.g*.[14–17]). However, as pointed out by [11], most published studies have focused on the most attractive candidate genes of all those identified, which generally encode detoxification enzymes. Thus, only part of the mechanisms potentially involved in NTSR has been scrutinised. Furthermore, perhaps because whole-transcriptome sequencing experiments remain expensive, the number and the diversity of the plants analysed are often limited. Furthermore, the vast majority of previous NTSR transcriptome studies compared resistant and sensitive plants, each from a different population (Table S1), thereby comparing plants with different genetic backgrounds. A few studies have avoided this drawback by comparing resistant and sensitive siblings from progenies derived from controlled crosses. However, this amounted to investigating the NTSR determinants present in a few, specific individual plants (*i.e*., the NTSR parent plants). In all cases, these experimental designs did not allow exploration of possible interpopulation variability in NTSR mechanisms. Last, validation is a crucial step in establishing the involvement of candidate genes in NTSR [14], and this has usually only been carried out using plants from the same population or line used to identify the candidate genes in the first place. The limitations of this population-centred approach are that i.) it does not allow exploration of the diversity of resistance genes present at the level of a weed species’ range, or at least across a broad geographical area, and ii.) it does not allow validation of genetic determinants of NTSR at the species level, so that they could be used for broad-scale resistance diagnostics.

In this study, we explored the diversity of mechanisms underlying constitutive and early herbicide-induced NTSR to ALS inhibitors that have evolved in ragweed across France. In a first step, we analysed six populations of ragweed from different French regions where NTSR to imazamox and/or tribenuron had evolved. By sequencing the transcriptomes of plants from geographically distinct populations and measuring the expression of candidate genes in a large number of plants, we were able to identify genes linked to NTSR and to deepen our knowledge of the evolutionary patterns of NTSR between different locations. In a second step, the value of a set of constitutive candidate NTSR genes as resistance predictors was assessed using a second independent and massive plant sampling.

## Material and methods

### Plant material

Six French ragweed populations where NTSR was identified as the only resistance mechanism to the ALS inhibitors imazamox and/or tribenuron in a previous work [12] were selected for this study (populations ARA2, ARA8, NAQ8, NAQ9, CVL5, and OCC13, Figure S1). *Ambrosia artemisiifolia* is not listed as an endangered or protected species, and collection of seeds from these populations had previously been permitted by the Directorate-General for Alimentation of the French Ministry for Agriculture within the framework of the governmental biological monitoring of the national territory that is part of axes 1 and 3 of the Ecophyto II plan for the reduction of pesticide use, and in particular within the framework of actions 5 of axis 1 (“Bulletin de santé du vegetal”) and 12 of axis 3 (Non-Intentional Effects of pesticides) (Reference texts: Article L.251–1 of the French Rural and Maritime Fishing Code and Circular CAB/C2009-002 of 4 March 2009). To ensure that any TSR mechanism was involved, systematic ALS gene sequencing of the resistant plants phenotyped was performed as described in [12]. ALS gene expression between resistant and sensitive plants from the different population was measured by qPCR as described in [12] to ensure no overexpression of the herbicide target (Figure S2). These populations were issued from distinct geographical origins (mean Euclidean distance = 249 kms) where NTSR to ALS inhibitors had evolved under different herbicide selective pressure and environmental conditions. The frequencies of plants resistant to imazamox and/or tribenuron in these populations are summarised in Table S2. Considering these frequencies, population ARA2 was used to seek genetic determinants of NTSR to imazamox while the other populations were used to seek genetic determinants of NTSR to tribenuron.

### Ragweed reference transcriptome

#### RNA extraction and PacBio sequencing

ALS inhibitors essentially act in active plant meristems [15] that are located at the apex of the shoots in dicotyledonous plants like ragweed [16]. We thus aimed at setting up a transcriptome including as many genes expressed in ragweed meristems as possible to be used as reference for the subsequent sequencing experiments. This implied sequencing the transcriptome of ragweed apical meristem before herbicide application (to further investigate constitutive NTSR), and at several time-points after herbicide application (to further investigate herbicide-induced NTSR). Transcriptome sequencing was performed from one single ragweed plant because of the high genetic variation observed among ragweed individuals and populations [17]. As vegetative propagation of ragweed is not feasible, we implemented a different strategy. Seedlings from the reference population P08 that exclusively consists of herbicide-sensitive plants were cultivated as described [12] until the four-leaf growth stage that is the stage recommended for ALS inhibitor spraying in the field. The apical bud and primordia of the fifth and sixth leaves were then collected using a disposable scalpel blade, and the cut was patched with a droplet of masking gum. This triggered the development of four offshoots (one per leaf axillary bud). When the two first leaves of each offshoot were fully expanded, a time-course experiment was conducted using imazamox at the maximum authorized French field rate (50 g imazamox per ha). Imazamox application was as described [12]. The apical bud and leaf primordia of one offshoot were sampled 2, 12, 24 and 48 h after application of the herbicide treatment. Each of the five samples per plant were collected in Eppendorf tubes containing two 3-mm diameter steel beads and placed in liquid nitrogen to avoid RNA degradation. Samples were stored immediately stored at -80°C until RNA extraction.

Total RNA was extracted using the Direct-zol RNA MiniPrep kit (Zymo research) following the manufacturer’s instructions. Total RNA concentration, sample quality and RNA integrity were checked using a NanoDrop spectrophotometer (LABTECH, Luton, UK) and Agilent 2100 Bioanalyzer System (Agilent, Waldbroon, Germany). Three plants which five RNA samples had both ratio values between 1.8 and 2.2 and a RIN > 7.5 were selected for mRNA purification. mRNA was purified with the AMBION Purist Mag Kit following manufacturer’s instructions. After mRNA extraction, all five mRNA samples per individual were pooled as an equimolar mixture. The three resulting pooled mRNA samples were sent to the GenoToul sequencing platform [18]. After quality control, the best-quality pooled mRNA sample was sequenced as 4 size fractions (≤ 2, 2–3, 3–6 and 5–10 kb) using a RSII sequencer (Pacific BioScience) with 2 Single Molecule Real Time sequencing (SMRT) cells per fraction size.

#### Reference transcriptome assembly and annotation

All reads that passed quality checks were used as starting material for de novo assembly. The first step was to assemble the reads obtained for the 8 SMRT cells by analysing the transcripts per size fraction using the PacBIO ‘RS_IsoSeq.1’ pipeline and removing strict redundancy. Redundancy was further suppressed based on a minimum identity of 99% and a maximum overhang of 50 nucleotides. Coding sequences in contigs were sought using FrameDP [19], implemented on Uniprot-Plants and the sunflower proteome database [20]. Blast2GO [21] was used for the annotation of the FrameDP predicted peptides using InterProScan [22] and i blast (BLASTx) hits versus the NCBI non-redundant database [23]. Last, BUSCO (Version 3, [24] was run to check the completeness of the assembled transcriptome.

### Whole-transcriptome sequencing

#### Plant material production

Five batches of phenotyped plant material (Table S3) were produced under the same experimental conditions at different period of time. Plants were grown in a greenhouse to ensure control of environmental conditions. Seeds were stratified and set to germinate as described [12]. Seedlings at the cotyledon stage were transplanted into 96-well trays, allowing individualisation of each plant. The potting soil used was a mixture of loamy soil, sand, perlite and peat (60%, 15%, 15% and 10% respectively). Seedlings were then transferred to the greenhouse (photoperiod of 16h, 20°C/15°C day/night, watering as needed). At the four-leaf stage, the apical bud and primordia of the fifth and sixth leaves from each seedling were collected as described above and stored at -80°C until total RNA extraction.

The apical bud of the seedlings in the four batches intended for the study of constitutive NTSR mechanisms (before treatment modality, BT) was collected 24 h before the application of the herbicide treatment. The apical bud of the batches of seedlings in the batch intended for the study of early herbicide-induced mechanisms was collected two hours after herbicide application (2HAT modality). Seedlings from population ARA2 were sprayed with imazamox and seedlings from populations ARA8, NAQ8, NAQ9, CVL5, OCC13 were sprayed with tribenuron. Both herbicides were applied as described [12], at their respective maximum field rate allowed in France.

In addition to the plants from the field populations of interest, each batch included ten treated plants from the reference population P08 as a check for the efficacy of the herbicide application, and ten water-sprayed plants from each of the field populations of interest and from the reference population as an untreated control. Apical buds from the control plants were collected under the same conditions as those from the plants intended for transcriptome sequencing to check that bud collection did not impact plant development. Four weeks after treatment, the phenotype (resistant or sensitive to the ALS inhibitor applied) of each sprayed plant was visually assessed as described [12], using untreated plants from each population as a reference.

#### Transcriptome sequencing

Four RNA sequencing (RNASeq) experiments were conducted on independent plant batches. Three aimed at investigating constitutive NTSR (BT modality), and one targeted early-induced NTSR (2HAT modality) (Table S4). Total RNA from each individual apical bud was extracted following the same procedure described for the reference transcriptome experiment (see above). Samples with the highest RNA concentration and a RIN > 8 were selected and pooled in equimolar mixtures according to the experimental design summarised in Table S4. Each batch of RNA pools was sequenced on an Illumina HiSeq 3000 or NovaSeq 6000 sequencer (Table S4). The 150-nucleotide pair-end sequences passing Illumina standard quality controls were mapped on the ragweed reference transcriptome using the glint aligner (version 1.0.rc12.826_833, 2018) configured to keep only matched sequences with the best alignment and no gaps in the alignment (maximum number of mismatches = 10 nucleotides, minimum length = 80 nucleotides, maximum distance allowed between each pair = 10,000). Reads were allowed to map to multiple contigs to avoid bias of analysis due to transcriptome redundancy. The total number of aligned reads per contig per sample was then computed and used to perform the differential expression analyses.

### Identification of candidate NTSR and/or herbicide response genes

#### Constitutive NTSR candidate genes

Counts from each of the three BT RNASeq experiments (RNASeq 1, 2 and 3a) were analysed independently. After removing lowly expressed contigs (counts < 50), data were normalised using the EDASeq package [25] from R [26]. The whole-transcriptome expression patterns of the different RNA pools were visualised using principal component analyses (PCAs) performed on count data normalized by the DESeq2 package with the FactoMineR package [27] and plotted with the FactoExtra package [28]. Identification of differentially expressed (DE) genes between resistant and sensitive pools was performed with the DESeq2 package [29] for each population and experiment independently to identify genes governing population-specific NTSR mechanisms. Difference in expression between resistant and sensitive pools were tested with a Wald test as described in [29] and p-values were corrected using the Benjamini and Hochberg procedure. These DE analyses were followed by Gene Ontology (GO) enrichment analyses using the topGO package [30] in R.

A set of constitutive NTSR candidate genes was selected from each population based on the magnitude of the difference in gene expression between resistant and sensitive pools (fold-change lower or higher than 2 on a log2 scale) and the adjusted *P*-value for multiple testing using the Benjamini–Hochberg method [31] (*P*-adj < 0.1). Contig annotation was also checked before selection but was not a discriminating criterion. We selected genes whose function may be involved in herbicide detoxification (*e.g.* cytochromes P450, glucosyl-transferases, ABC-transporters…) as well as resistance proteins, transcription factors, regulatory proteins and proteins with unknown function.

#### Herbicide-induced response in resistant and sensitive plants

Counts from the 2HAT modality were analysed in two steps and independently for each population. In the first step, genes DE between the resistant and sensitive pools in each population were sought as described above. The DE gene lists generated were compared among populations and to those obtained for the BT modality within each population. In a second step, the early response of each phenotype to herbicide application was assessed by seeking the genes DE between BT and 2HAT in the resistant pools or in the sensitive pools in each population independently. GO enrichment analyses were then conducted as previously to characterise the early herbicide response of each phenotype within each population.

### Validation of constitutively expressed candidate NTSR genes

Constitutive NTSR candidate genes were selected on the basis of the expression levels measured by RNAseq in a relatively small number of plants organised in pools (Table S4). Within-population validation of the link between gene expression level and plant phenotype was performed using reverse-transcription followed by Fluidigm® qPCR (RTqPCR) on individual plants from batch 1, 2 and 3 (Table S3). cDNA was synthesized from 1 µg of total RNA extracted from individual plants using the Quantitect® Reverse Transcription Kit (Qiagen, Courtaboeuf, France). Gene relative expression level was measured in individual plants according to the 2^∆∆Ct^ method [32]. Expression data was normalised using three reference genes (ubiquitin, filamin, and GAPDH) and three standard RNA samples from the reference population P08. The reference genes had been validated in a preliminary experiment where the relative expression of 10 candidate reference genes was measured by RTqPCR on cDNAs from 250 resistant or sensitive plants in four ragweed populations. The candidate reference genes had been selected based on the literature [33, 34] and among genes showing a stable expression in our first RNASeq experiment (RNASeq 1, Table S4). The most stable reference genes were identified using the Genorm [35] and Normfinder [36] algorithms available in the “selectHKs” function of the NormqPCR package [37] for R and the “tidy_normfinder” function available at [38].

Fluidigm® qPCR was performed at the platform Gentyane (INRAE, Clermont-Ferrand, France; [39]) following the Fluidigm® recommended procedure and starting from 5 ng/µL cDNAs diluted in TE low EDTA (10mM tris–HCl, 0.1 mM EDTA, pH8). A ½ point dilution range was performed from a pool of ten samples to verify the efficiency of primers. After a preamplification step and exonuclease treatment, a mix was prepared for each of the primer and cDNA plates. Primers and cDNAs were diluted four times in the mix prepared. IFC Dynamic Arrays 96.96 (Fluidigm®) were then prepared manually from the diluted primers and cDNA. Runs were performed on a Fluidigm BioMark HD Real-Time PCR system (IFC controller HX). The expression of constitutive NTSR candidate genes was measured in all individual plants (biological replicates) in batches 1, 2 and 3a (Table S3), including the plants used for RNAseq experiments 1, 2 and 3a. The expression of 91 candidate genes identified in RNAseqs 1 and 2 was first measured in all plants in batches 1 and 2 (Figure S3). Twenty-seven candidate genes were retained. Their expression and that of 30 additional candidate genes identified in RNASeq 3a was measured in all plants in batch 3a (Figure S3). All Fluidigm® qPCR data was pooled after verifying that gene expression levels in the three standard samples were identical in all runs. Genes linked to NTSR were identified within each population as the candidate genes with a significantly different relative expression level between resistant and sensitive plants, as checked by a Wilcoxon rank test.

### Prediction of plant phenotypes based on constitutive candidate contig expression patterns

Identification of genes with constitutive expression patterns related to NTSR would have a direct application for resistance diagnosis. This diagnosis is all the more challenging as the genetic determinants of NTSR are polygenic [13]. In this section, using the RTqPCR thanks to the data collected previously for a very large number of NTSR candidate genes, we sought to identify the optimum gene combinations for predicting plant phenotype with the greatest accuracy. A two-step approach was conducted with 1) construction of the predicting models based on the expression data generated previously and 2) test of the models on expression data obtained for a new sampling of plants (batch 4, Table S3).

Firstly, linear discriminant analysis with LOOCV (Leave-One-Out Cross Validation) resampling, implemented using the “train” function in the Caret R package [40], was conducted using the expression data obtained by Fluidigm® RTqPCR for all candidate NTSR genes from all plants in each of the populations ARA2, ARA8 and NAQ8 collected in batches 1, 2 and 3a (Table S3). All candidate genes were ranked in each population according to their ability to discriminate plant phenotypes. The applied objective of this analysis was to evaluate our ability to detect resistance based on the expression pattern of a small set of candidate genes, so that RTqPCR-based diagnosis is feasible. We thus decided to seek the minimum combination of genes enabling to diagnose resistance with the greatest accuracy starting with the ten top-ranked genes in each population. Linear discriminant analysis and LOOCV resampling was iterated eight times, removing the gene with the lowest contribution to phenotype prediction at each step. This approach allowed us to select the combination of genes that best predicted the phenotype within each population based on the model accuracy (*i.e*., its sensitivity that is its ability to predict that a resistant plant is resistant, and its specificity that is its ability to predict that a sensitive plant is sensitive).

Secondly, we measured the expression levels of the genes composing the previously established gene combinations on an independent batch of plants (batch 4, Table S3). Accuracy of the gene combination of each population to predict the phenotype with this new expression data was evaluated with the “test” function of the Caret package. Expression data were obtained by classical RTqPCR performed in 384-well plates on cDNAs diluted 24 times in RNA/DNA free water. A ½ point dose range was performed to check primers efficiency. Plates were manually prepared by adding 3 µL diluted cDNA and 7 µL mix per well (one primer tested per plate). Runs were performed on a QuantStudio™ real-time PCR system (AppliedBiosystem). Gene relative expression level was measured in individual plants according to the 2^∆∆Ct^ method as described before. Difference in expression between resistant and sensitive plants in this new sampling for each gene that compose the different gene combination established was additionally evaluated with a Wilcoxon rank test.

## Results

### AMBELbase, a high-quality reference transcriptome resource for common ragweed

A reference transcriptome for ragweed was assembled from a pool of mRNA extracted from apical buds and leaf primordia collected on a single ragweed plant before and at several time-points after application of the ALS inhibitor imazamox. The 109,560 PacBio quality reads obtained totalised 225.1 Mb. After suppressing redundancy, 51,242 contigs ranging in size from 301 to 11,889 nucleotides with a N50 value of 2,349 nucleotides were obtained (110.5 Mb in total) and subjected to coding sequence search and functional annotation. BUSCO analysis estimated our reference transcriptome to be 80.2% complete with 34.2% complete and single-copy orthologs, 46% complete and duplicated orthologs (likely because of ragweed high heterozygosity [17], 2.6% fragmented orthologs and 17.2% missing orthologs out of a total of 1,440 ortholog groups searched. The contigs and their predicted peptides are available at [41].

### Identification of constitutive NTSR candidate genes

**Fig. 1:**
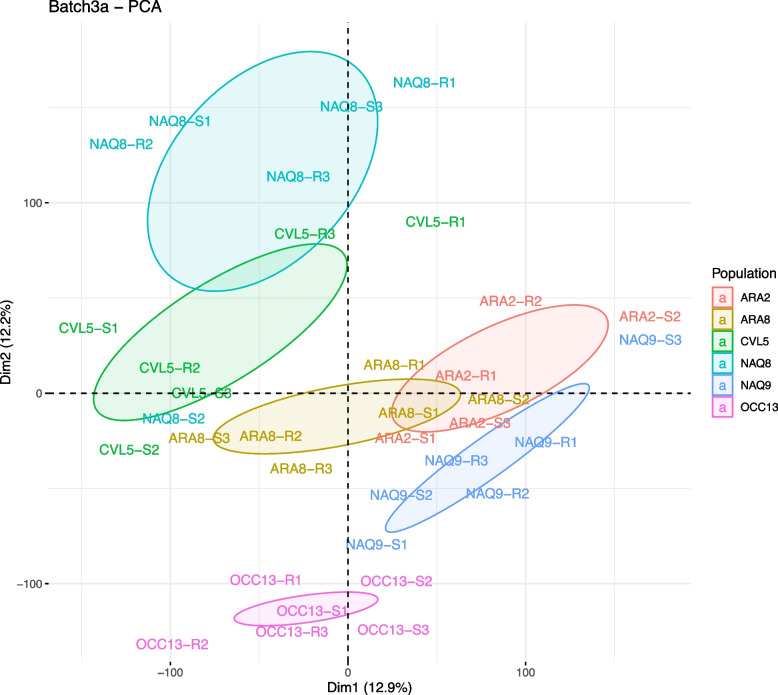
Principal component analysis of the global expression profiles of plant RNA pools in batch 3a (before treatment (BT) modality, field populations ARA2, ARA8, NAQ8, NAQ9, OCC13 and CVL5) obtained from experiment RNASeq 3a. Each pool is indicated by a label showing the name of the population, the phenotype of the plants in the pool, and the pool number (i.e., the replicate number for one given population and phenotype). Circles represent the 95% confidence interval around the barycentre of the samples of each population. Plants in population ARA2 pools are resistant or sensitive to imazamox; plants in the others pools are resistant or sensitive to tribenuron

**Fig. 2:**
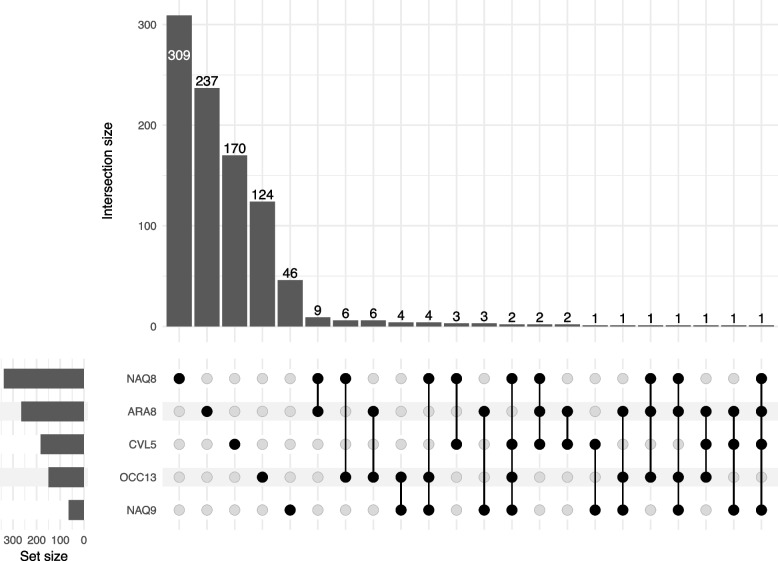
Number of genes DE in one or several of the five populations with plants resistant to tribenuron

PCAs on transcriptome expression data were performed independently for each of the three ‘constitutive’ RNASeq experiments to visualize relationship between plant pools (See Fig. 1 for experiment RNASeq 3a, which included all six populations, and Figure S4 for the other RNASeq experiments). The first two axes of the PCAs explained together 25.1%, 32.3 and 79.8%, respectively, of the variance present in the expression data from RNAseq3a, 2 and 1. Overall, the plant pools were grouped according to their population of origin rather than according to their resistance phenotype (Fig. 1, Figure S4). Given the difference in the overall expression profile between each population, constitutive NTSR candidate genes were sought in each population independently (*i.e.,* gene expression was compared between the resistant and the sensitive plant pools within each population) and the lists of genes obtained were compared among populations. In population ARA2 with imazamox-resistant plants, a total of 3,616 genes with significant DE were identified. In the five other populations with tribenuron-resistant plants, a total of 995 DE genes were identified (270 in population ARA8, 181 in population CVL5, 337 in population NAQ8, 64 in population NAQ9 and 141 in population OCC13). No DE gene was common to all populations nor to the five populations with tribenuron-resistant plants, but some genes were DE in two to four populations (Fig. 2, Table S6). Yet, most of the DE genes identified were specific to one population.

GO enrichment analyses were performed on DE genes identified in each population and did not identify any Biological Process (BP) or Molecular Function (MF) common to all populations studied (Table S7). Terms related to gene expression regulation or plant defence and secondary metabolism processes were common to four or three populations, respectively (Table S7).

Overall, although similarities existed among some populations, no DE genes, BP or MF common to all populations or to the five populations with tribenuron-resistant plants were identified. Candidate NTSR genes were therefore selected in each population independently for further validation. A total of 121 candidate genes DE between resistant and sensitive pools in at least one population were retained (45 from population ARA2, 12 from population ARA8, 17 from population OCC13, 42 from population NAQ8, nine from population CVL5, and three from population NAQ9, Table S8), of which nine were common to several populations.

### Identification of herbicide-induced NTSR candidate genes

As in ‘constitutive’ RNASeq experiments, a strong population effect was observed in the ‘induced’ RNASeq experiment (2HAT modality) (Figure S5). Overall, no gene was significantly DE between all resistant and all sensitive pools 2 h after herbicide application (2HAT). Genes DE between resistant and sensitive pools 2HAT were thus sought in each population individually (Table S9). Comparison of the lists of genes identified in each population showed that no genes were commonly DE in the six populations or in the five populations with tribenuron resistant plants. One gene was DE in four populations, six in three populations and 33 in two populations (Table S10). GO analysis independently performed on each list showed that no BP or MF were common to all the populations (Table S11).

Comparison of the list of genes constitutively DE (resistant BT *vs*. sensitive BT) and DE following herbicide application (resistant 2HAT *vs*. sensitive 2HAT) in each of the six populations indicated that one to 13 genes were DE between resistant and sensitive pools both before and after treatment (Table S9). Of the 121 constitutive NTSR candidate genes previously selected, 14 were also significantly DE between resistant pools and sensitive pools 2HAT (Table 1). Six of the candidates with a higher expression in resistant pools before treatment in one population and after treatment in another were predicted to code for proteins that could be directly involved in NTSR (two cytochromes P450 in families 71 and 72, one oxidoreductase, one dehydrogenase, one germacrene synthase, and one protein induced by the jasmonic acid pathway).

**Table 1:** Candidate genes DE between resistant and sensitive pools in one population both before and after treatment in RNAseq experiments. RBT/SBT, resistant *versus* sensitive pools before treatment; R2HAT/S2HAT, resistant *versus* sensitive pools 2 h after treatment

			**Before treatment (BT)**			**2 h after treatment (2HAT)**					
**Accession**	**code**	**Description**	**Population**	**Log2FC RBT / SBT**	**padj**	**Population**	**Log2FC R2HAT / S2HAT**	**padj**	**Population**	**Log2FC R2HAT / S2HAT**	**padj**
AMBELPO8A3r1t_44453	CYP71-2	cytochrome P450, family 71, subfamily B	NAQ8	12.06	4.73E-04	OCC13	15.81	1.03E-02	ARA8	10.28	3,19E-04
AMBELPO8A3r1t_38863	OX	probable quinone oxidoreductase	NAQ8	1.97	1.55E-03	CVL5	-4.10	1.78E-02	ARA8	7.06	5.42E-04
AMBELPO8A3r1t_46903	Jsm	23 kDa jasmonate-induced -like	ARA8	24.36	1.42E-09	NAQ8	10.87	2.97E-02			
AMBELPO8A3r1t_26309	GMC	germacrene D synthase	NAQ8	16.36	6.06E-05	ARA2	23.02	1.85E-07			
AMBELPO8A3r1t_27615	CYP72-2	cytochrome P450/ CYP 72A	NAQ8	7.80	5.06E-11	ARA8	10.04	1.83E-05			
AMBELPO8A3r1t_48133	Dhs2	alcohol dehydrogenase-like 7	OCC13	7.81	5.12E-16	NAQ9	12.81	3.45E-02			
AMBELPO8A3r1t_40047	HDL	bifunctional epoxide hydrolase 2-like	ARA8	2.25	8.96E-06	NAQ8	-2.39	1.83E-03			
AMBELPO8A3r1t_31733	Tptr	uncharacterized transporter	ARA2	6.57	2.80E-25	NAQ8	-3.86	8.89E-04			
AMBELPO8A3r1t_29358	GT-4	7-deoxyloganetin glucosyltransferase-like	ARA2	5.32	1.47E-27	OCC13	-2.25	4.12E-02			
AMBELPO8A3r1t_42447	GST-1	glutathione S-transferase T3-like	OCC13	1.99	8.71E-02	OCC13	-2.38	1.87E-02			
AMBELPO8A3r1t_13971	MeT	homocysteine S-methyltransferase 2 isoform X1	OCC13	5.72	5.36E-14	OCC13	-5.76	1.76E-02			
AMBELPO8A3r1t_26652	GT-5	UDP-glycosyltransferase 73C3-like	ARA2	7.78	1.18E-26	OCC13	-2.43	3.40E-02			
AMBELPO8A3r1t_43085	CYP71-3	cytochrome P450/ CYP 71B	ARA8	22.53	2.24E-02	ARA2	-15.21	3.71E-02			
AMBELPO8A3r1t_44569	Dhs3	glycerate dehydrogenase	ARA8	9.12	1.2E-08	ARA2	-18.66	1.71E-07			

### Differences in gene expression caused by herbicide application in resistant pools or in sensitive pools

The response of each phenotype to treatment (resistant 2HAT versus resistant BT and sensitive 2HAT versus sensitive BT) was assessed within each population. The number of genes DE after herbicide application ranged from 795 to 4287 in resistant pools and from 3734 to 10,174 in sensitive pools, depending on the population (Table S12). We then sought genes DE after treatment in resistant pools (resistant 2HAT versus resistant BT) common to multiple populations. None was DE after treatment in all resistant pools from all populations. Eight were significantly DE in three populations, and 123 in two populations.

**Fig. 3:**
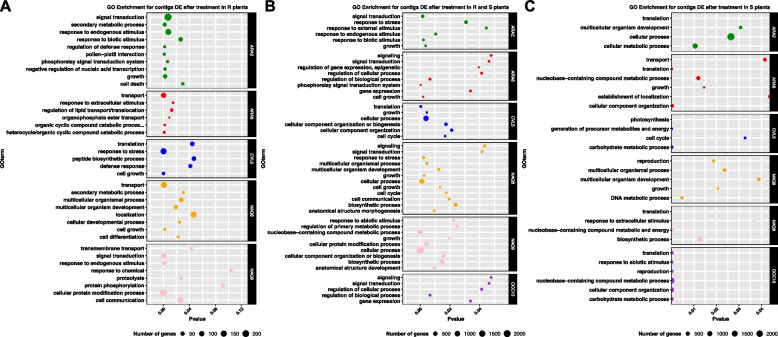
Result of GO enrichment analysis for genes DE after herbicide application in each phenotype in the six populations studied. The BP terms displayed are significant at the elim-Fisher test (*p*-value < 0.05) and represent at least 1% of the DE genes in the population of interest. **A** terms associated with DE genes specific to resistant plants, **B** terms associated with DE genes shared by resistant and sensitive plants, **C** terms associated with DE genes specific to sensitive plants

A GO enrichment analysis identified BPs enriched in both resistant and sensitive plants in five of the six populations, mostly related to stress perception and stress response (response to biotic stimulus, response to external stimulus, response to endogenous stimulus, response to stress) (Fig. 3). They also included regulatory pathways (regulation of gene expression, epigenetic, phosphorelay signal transduction system, signal transduction, signalling, cell communication) within four of the six populations studied (Fig. 3). In resistant plants specifically, BPs related to stress perception, regulatory changes and plant secondary metabolism were enriched. This concerned transport (transport, organophosphate ester transport, transmembrane transport) for populations NAQ8, NAQ9 and ARA8, the catabolism of complex, organic molecules (heterocycle/organic cyclic compound catabolic process) in population ARA8, protein modifications (protein modification process, cell protein modification) in populations OCC13 and NAQ9. In sensitive plants specifically, BPs mostly associated with plant primary metabolism (growth, cell cycle, photosynthesis, reproduction) (Fig. 3) were enriched in all populations. Protein translation was also enriched after treatment in four populations.

### Validation of constitutive NTSR candidate genes

**Fig. 4:**
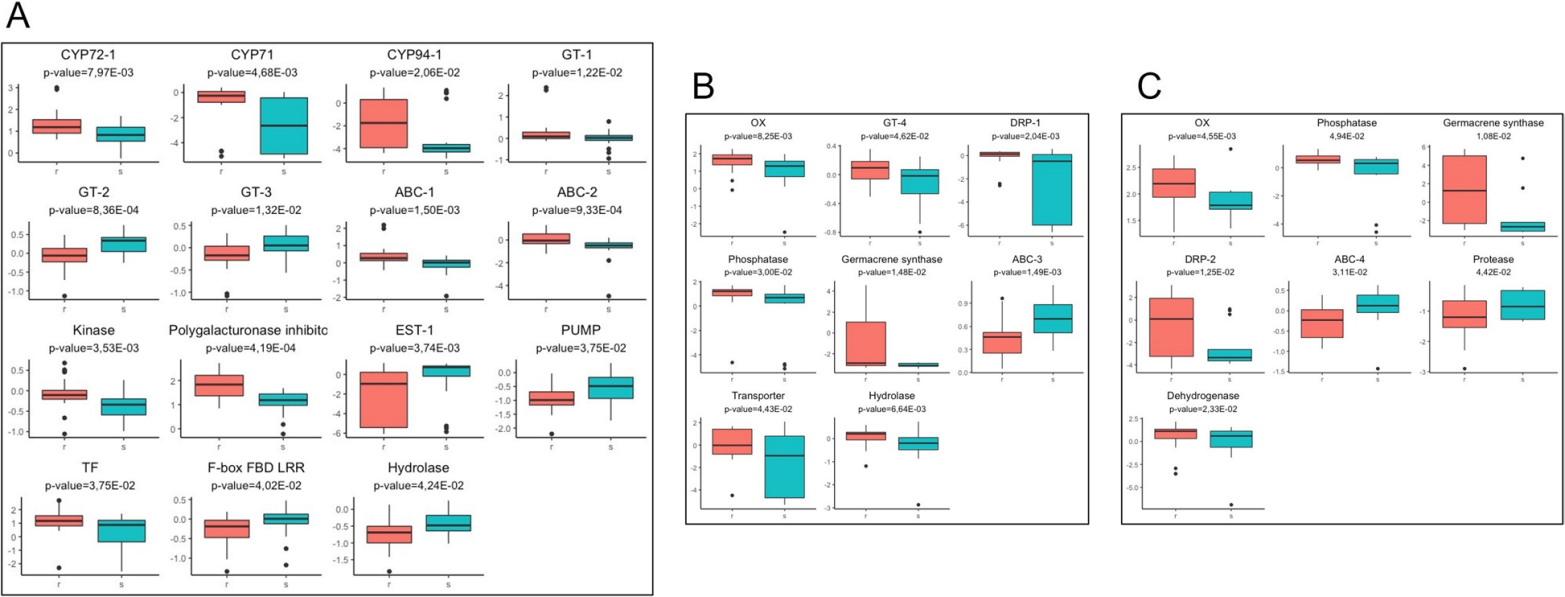
Relative expression levels measured in individual plants resistant (red) or sensitive (blue) to imazamox (population ARA2, **A**) or tribenuron (populations ARA8, **B**, and NAQ8, **C**) for the candidate genes validated in each population

Relative expression of the 121 constitutive candidate genes selected (Table S8) was measured in the 220 individual plants in batches 1, 2 and 3a that included the 149 plants sequenced as pools in the RNAseq experiments and 71 additional plants (see details in Table S3). Of the 121 candidate genes, 28 (23%) identified in one of the populations ARA2, ARA8 or NAQ8 were confirmed to be significantly DE between resistant and sensitive plants (Fig. 4). No candidate gene identified in populations OCC13, CVL5 or NAQ9 was validated.

In population ARA2, 15 genes were significantly DE between the resistant and the sensitive plants (Fig. 4A). They included two ABC transporters (ABC-1 and ABC-2), one resistance protein (DRP-1), one LRR receptor (F-box FBD LRR), one polygalacturonase inhibitor (PGIP), and three cytochromes P450 (CYP71-1, CYP72-1, and CYP94) from families 71, 72, and 94 that showed significantly higher relative expression levels in resistant plants compared to sensitive ones. One efflux pump (PUMP), one hydrolase (HDL) and one esterase (EST-1) showed significantly lower relative expression levels in resistant plants compared to sensitive ones. In population ARA8, eight genes were significantly DE between the resistant and the sensitive plants (Fig. 4B). They included one resistance protein (DRP-1), one phosphatase (Phs), one oxidoreductase (OX), one glycosyltransferase (GT-3), one hydrolase (HDL), one transporter (TBC), and one enzyme involved in terpene synthesis (GMC) that showed significantly higher relative expression levels in resistant plants compared to sensitive ones. One ABC transporter (ABC-3) showed significantly lower relative expression levels in resistant plants compared to sensitive ones. In population NAQ8, seven genes were significantly DE between the resistant and the sensitive plants (Fig. 4C). They included one resistance protein (DRP-2), one oxidoreductase (OX), one phosphatase (Phs), one dehydrogenase (DH), and one enzyme involved in terpene synthesis (GMC) that showed significantly higher relative expression levels in resistant plants compared to sensitive ones. One ABC transporter (ABC-4) and one protease (PROT) showed significantly lower relative expression levels in resistant plants compared to sensitive ones. Five genes were validated in two populations: GT-3 and HDL in populations ARA2 and ARA8, and OX, GMC and Phs in populations ARA8 and NAQ8.

### Predicting plant phenotype using expression data of a combination of NTSR candidate genes

**Fig. 5:**
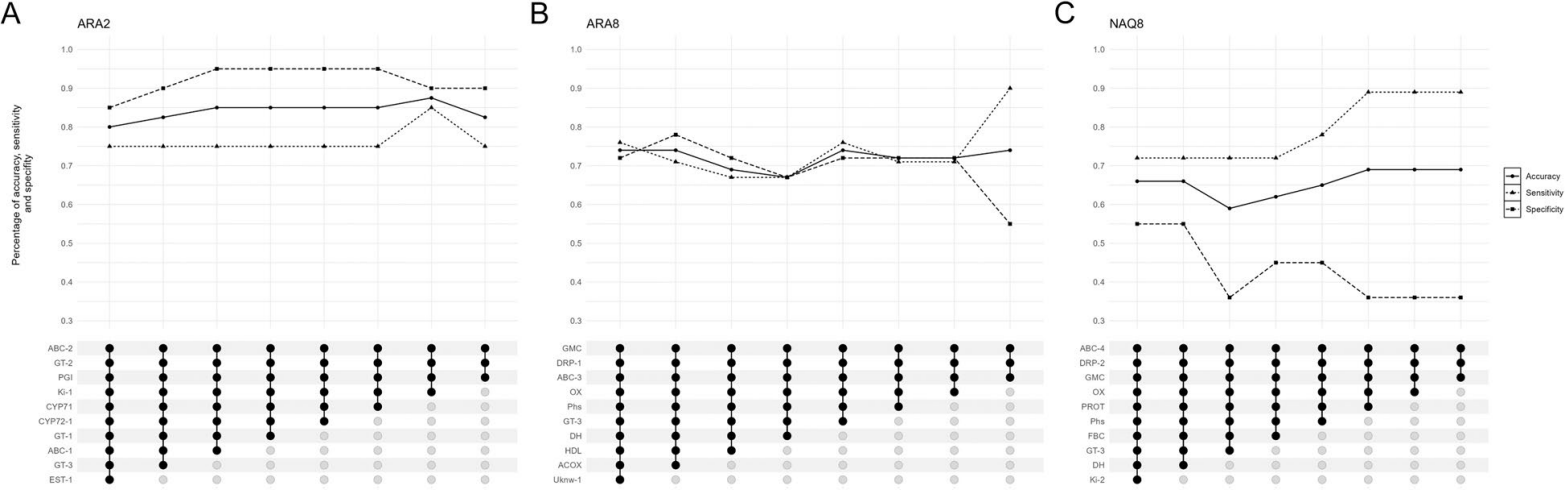
Variation of the accuracy (circle), sensitivity (triangle) and specificity (square) of the eight models designed to predict plants phenotype thanks to expression levels in ARA2 (**A**), ARA8 (**B**) and NAQ8 (**C**) populations

Identification of genes with constitutive expression profiles related to NTSR would have direct application for resistance diagnosis, but requires the identification of all the genes involved in the construction of this resistance. Therefore, we investigated whether a small set of candidate genes, rather than a single gene, would better predict the phenotype. All 121 constitutive candidate NTSR genes for which expression data was obtained in individual plants were ordered according to their respective contributions to plant phenotype prediction in each of populations ARA2, ARA8 and NAQ8 using linear discriminant analysis with LOOCV of their relative expression levels (Table S5) and the ‘top ten’ in each population were selected. The respective sets of ten genes were then used in a LOOCV resampling with eight iterations to assess the relevance of plant phenotype prediction based on the expression data of a gene combination (Fig. 5). Statistics for each model are detailed in Table S13. Overall, there was no clear trends in the relationship between the number of genes included in the model and phenotype prediction accuracy (Fig. 5).

In population ARA2, the accuracy of the models ranged from 80% to 87.5%, its sensitivity from 75 to 85% and its specificity from 74 to 95%. The gene combination maximising accuracy for this population was PGI + GT-2 + ABC-2 + Ki-1 (accuracy = 87.5%, sensitivity = 85%; specificity 90%) (Fig. 5A).

In population ARA8, the accuracy of the different models ranged from 67 to 74%, its sensitivity from 67 to 90% and its specificity from 55 to 78%. The gene combination maximising accuracy for this population was ABC-3 + DRP-1 + GMC + OX + Phs + GT-3 (accuracy = 74%, sensitivity = 76%, specificity = 72%) (Fig. 5B).

In population NAQ8 the accuracy of the different models ranged from 59 to 69%, its sensitivity from 72 to 89% and its specificity from 36 to 55%. The gene combination maximising accuracy for this population was GMC + DRP-2 + ABC-4 (accuracy = 69%, sensitivity = 89%, specificity = 36%) (Fig. 5C).

The respective models including the optimal gene combination identified in each of the three populations ARA2, ARA8 and NAQ8 were tested on a new and independent batch of plants (93 plants from population ARA2, 129 from population ARA8 and 114 from population NAQ8; batch 4, Table S3). This batch did not include any plant used to establish the gene combinations in question, so that the relevance of the genes included in the models could be assessed for NTSR diagnosis.

In population ARA2, the accuracy of the model was 61% for the plants in batch 4 (-26.5% compared to the plants in batches 1, 2 and 3a used to build the model), with a sensitivity of 86% (+ 1%) and a specificity of 42% (-48%). For population ARA8, the accuracy obtained was 46% (-28%) with a sensitivity of 70% (-6%) and a specificity of 26% (-46%). For population NAQ8, the accuracy obtained was 48% (-21%) with a sensitivity of 77% (-12%) and a specificity of 22% (-14%).

## Discussion

Identifying the genes underlying NTSR to herbicides without a priori knowledge of the resistance mechanisms and considering the genetic diversity of a weed species requires the use of a combination of high-throughput sequencing and careful experimental design. In this study, we analysed a set of geographically diverse field populations of ragweed where resistance to ALS-inhibiting herbicides had evolved under the selective pressure of recurrent herbicide applications. We identified a wide variety of constitutive or herbicide-induced genes potentially involved in NTSR to ALS inhibitors. These genes were mostly population-specific.

### A wide variety of NTSR mechanisms evolved among and within ragweed populations in France

ALS inhibitors are leaf-applied herbicides that essentially act at the level of the aerial meristems within a few hours of application [15]. To study the constitutive and early-induced mechanisms of NTSR to ALS inhibitors in ragweed, we established a transcriptome of the vegetative and aerial parts of a ragweed plant, including samples taken before and after treatment. This transcriptome showed a completeness of 80%, with a high number of duplicated BUSCO orthologs that is most likely a consequence of the naturally high heterozygosity of ragweed [17]. Our ragweed transcriptome is the most comprehensive resource available to date for ragweed transcriptomic studies. It was used as a reference for transcriptome sequencing experiments conducted on six ragweed field populations.

Transcriptome sequencing of plants from the different population revealed that expression profiles were specific to each population, regardless of their geographical origin or their resistance profile to imazamox or tribenuron. None of the identified constitutive or induced NTSR candidate genes were common to all populations studied, and very few (0.007%) were shared by several populations. Furthermore, the constitutively DE genes identified in the different populations did not necessarily contribute to the same biological processes or molecular functions. This suggests that a variety of metabolic pathways specifically activated in resistant plants may be involved in NTSR in ragweed. The diversity of NTSR mechanisms also appears to be considerable within populations: combinations of constitutive NTSR candidate genes initially found to be optimal for predicting plant phenotypes in one plant sampling from one population did not accurately predict the phenotype of plants in another, independent sampling from the same population. Additional results (Table S14) showed that genes with expression patterns linked to NTSR in one sampling from a given population were not necessarily linked to NTSR in a second sampling from the same population. This suggests that our RNA sequencing experiments merely captured a subset of the mechanisms involved in NTSR in each of the ragweed populations investigated, and that these mechanisms are thus most likely highly diverse within a population. This tremendous diversity may be due to the unprecedented number of plants used for ragweed transcriptome sequencing experiments (from 90 to 129 plants per population) compared to other similar studies (Table S1, mean = 24 ± 56 plants). Overall, our results revealed a non-redundant evolution of NTSR to ALS inhibitors in common ragweed populations. Independent evolution among populations may result from local adaptation in response to different selection pressures [42]. Indeed, [10] showed through recurrent selection that evolution of NTSR in ragweed populations is rapid and involves partially divergent mechanisms depending on the selection pressure exerted. The ragweed populations in our study were collected in different regions in France. As cropping systems, crop rotations and therefore inherent agronomic practices, such as herbicide spraying programs, are specific to each field and each grower, these populations had likely been subjected to different selection pressures and agronomical practices.

Furthermore, ragweed populations are characterized by a high genetic diversity at neutral markers [17]. This suggests large effective population sizes and a high amount of standing genetic variation at adaptive genes, which is expected to foster ragweed adaptation [43] and herbicide resistance evolution [44, 45]. The high genetic diversity of ragweed may also have facilitated the evolution of multiple NTSR mechanisms among and within populations, as observed for TSR in this species [12]. A diversity of xenobiotic metabolism pathways, constitutive and/or induced in answer to herbicide treatment, had also been observed in previous studies investigating the evolution of resistance in several weed populations. In their work, [46] demonstrated through recurrent selection on *Alopecurus myosuroides* that plants from the same population subjected to different selection pressures developed distinct NTSR mechanisms. On the same species, [47] showed similar results by comparing transcriptome and QTLs from two populations where NTSR had independently been selected for. [48] indicated that the evolution of NTSR to glyphosate in different populations of *Ipomoea purpurea* followed an evolutionary pattern that was divergent for several loci. All these examples are of ‘recent’ evolution of resistance, as is the case for the resistance of ragweed to ALS inhibitors in France that is still emerging and remains confined to a relatively limited number of fields [12]. Yet, when resistance has evolved over a period long enough that ‘primary’ NTSR mechanisms have been ‘filtered’ for resistance patterns and/or associated fitness cost in weed populations, redundant evolution can be observed (*e.g*., 50). It remains to be seen whether this will be the case in ragweed.

### NTSR, a complex interplay between multiple protective and regulatory genes

The genes associated with NTSR identified in our study allowed to predict resistance in only a subset of the plants in the populations studied, indicating that some, but not all, of the genes presumably involved in NTSR were identified. This finding highlights the complexity and the diversity of NTSR mechanisms in ragweed. It is consistent with the diversity of genes and gene families linked to NTSR observed in previous studies among species, among herbicides, and among populations of the same species (Table S15). Yet, many of the functions assigned to the candidate genes identified in ragweed were consistent with existing knowledge about the mechanisms endowing NTSR [13]. Describes NTSR as a set of mechanisms constitutive and/or induced by herbicide stress that involve the expression of “protective” and “regulatory” genes. Both categories of genes were identified in our study. Regulatory genes are involved in signalling and regulation of the stress response [13]. In response to herbicide application, the stress signal is carried to regulatory genes and triggers regulatory cascade(s) that, in turn, activate or enhance herbicide metabolization and enable the plant to survive [49]. GO enrichment analysis in our study suggested that genes or pathways regulating gene expression were DE in a constitutive and/or induced manner in resistant plants in several populations. Protective genes are involved in the four-step degradation of xenobiotics, including herbicides [50], that briefly consist into (1) increasing the solubility of the herbicide molecule (e.g., by reactions catalysed by cytochromes P450); (2) conjugation with water-soluble metabolites (e.g., through the action of gluco/glyco/gluthation/amino acid transferases); (3) transport into the vacuole or the cell walls (e.g.*, *via ABC transporters); (4) final degradation. Protective genes are the genes most commonly reported in NTSR studies, including studies addressing ALS inhibitors (Table S15). Our study is no exception, and GO enrichment analyses identified molecular functions related to oxygenase, oxidoreductase and transmembrane transport activities in ragweed. Also, 16 ragweed candidate genes (e.g. CYP72-1, CYP94, GT-1 to 4, ABC-1 to 4) may be directly related to herbicide degradation [50]. Furthermore, several of our candidates showed strong homology with NTSR candidate genes identified in other studies. CYP72-1 identified in ragweed population ARA2 was the most likely ragweed homolog of a CYP72 gene DE between glufosinate-resistant and glufosinate-sensitive plants from *A. palmeri* [51] (77% homology, e-value 5.6E-07) and of a CYP72 gene DE between *Myosoton aquaticum* plants resistant or sensitive to the ALS inhibitor tribenuron [52] (77.4% homology, e-value 9.74E-11). This CYP72 cytochrome P450 family was also involved in diclofop detoxification in *L. rigidum* [53] and in NTSR to ALS inhibitors in *E. phyllopogon* [54]. CYP71-1 identified in ragweed population ARA2 belongs to a P540 family that has been shown to play a role in the detoxification of ALS inhibitors in in soybean and wheat [55, 56] and in the weed *Descurainia Sophia* [57]. CYP94 identified in ragweed population ARA2 showed strong homology to a CYP94 gene linked to resistance to ALS inhibitors in *E. phyllopogon* [54] (79% homology, e-value 0.005) and to resistance to glufosinate in *A. palmeri* [51] (70.8% homology, e-value 2.08E-25). ABC-1, ABC-3 and ABC-5 identified respectively in ARA2, NAQ8 and OCC13 populations, was homolog of two genes (c50054_g1 and c39205_g1) identified as DE between *Myosoton aquaticum* plants resistant or sensitive to the ALS inhibitor tribenuron [52] (from 71.61% to 74.06% homology, min e-value 3.05e-08). GT-5 identified in ARA2 population showed homology with one gene identified as DE between resistant and sensitive plants to mesosulfuron of *Aegilops tauschii* [58] (84% homology, e-value 1.31e-09).

Our study was designed to allow screening a wide variety of NTSR mechanisms without making any assumptions related to their function. Therefore, we also considered and validated candidate genes whose direct involvement in herbicide degradation or regulation of the stress response is not obvious. This is the case for two disease resistance proteins (DRP1 and 2) and one enzyme involved in terpene synthesis (GMC, a germacrene synthase). Disease resistance proteins are involved in the response of plants to pathogenic stress or disease [59], and previous studies also associated an increased expression of disease resistance proteins in weed species with herbicide resistance [46, 60, 61]. While germacrene synthase is mostly associated with the response of plants to insects or microorganisms [62], a germacrene-D-synthase was identified in glyphosate-resistant plants of *Ipomoea purpurea* [63]. Such genes may well be NTSR markers (i.e., genes linked to NTSR pathways without being directly involved).

## Conclusion

Our study highlights the complexity and the diversity of NTSR mechanisms that can evolve in a weed species in response to herbicide selective pressure. In a previous work [12], we uncovered a tremendous diversity of TSR mutations having evolved in French ragweed populations. The present study complements and extends these findings by demonstrating that ragweed also has the capacity to harness a diversity of NTSR mechanisms that similarly varies within and among populations. Our work adds to the current body of evidence supporting the hypothesis of primarily non-redundant evolution of NTSR to herbicides in weeds, demonstrating the ability of plants to adopt different evolutionary pathways in response to herbicide-induced selective pressures. Furthermore, our results imply that common ragweed populations in arable fields have ample standing genetic variation allowing for rapid evolution of not only resistance to herbicides, but also, most likely, to other drastic environmental or human-driven selective pressures.

### Supplementary Information

**Additional file 1: Table S1.** Review of previous studies and their experimental design aimed at identifying genetic determinisms of NTSR using a transcriptomic approach. **Table S2.** Frequencies of plants resistant to imazamox and tribenuron in the six ragweed populations used in this study (source: Loubet et al., 2021). **Table S3.** Number of individual plants per population and per phenotype in the five batches produced for this study. **Table S4.** Experimental design of the four RNA sequencing experiments. **Table S5.** Ranking of all candidate genes for the ability of their expression level to discriminate phenotype obtained by linear discriminant analysis for each of the three populations, ARA2, ARA8 and NAQ8. By default, the top 20 variables are ranked by the caret package. The top 10 were selected for further analysis. **Table S6.** Genes constitutively DE between resistant and sensitive plant pools (Log2FC and associated adjusted p-value) identified in 4, 3 or 2 populations with plants resistant to tribenuron. Genes with a higher or lower expression in resistant plants compared to sensitive plants are highlighted in orange or in green, respectively. Genes with no DE are highlighted in green. **Table S7.** Biological processes and molecular functions associated with DE genes significantly enriched in at least 2 populations (elim Fisher test). **Table S8.** DE genes selected for validation of their potential link with NTSR (genes independently selected in more than one population are highlighted in green). **Table S9.** Number of genes DE  between resistant and sensitive plant pools in each population before (BT) and/or 2 hours after (2HAT) treatment. **Table S10.** Genes significantly DE 2HAT between resistant and sensitive plant pools identified in at least 2 populations. Genes with a higher or lower expression in resistant pools compared to sensitive pools are highlighted in orange or in green, respectively. **Table S11.** Shared biological processes (BPs) or molecular functions (MFs) associated to genes DE between resistant and sensitive plants 2HAT in at least 2 populations (elim Fisher test). **Table S12.** Number of genes DE  before and after treatment for each phenotype and each population. **Supplementary Table S13.** Complementary statistics obtained in the establishment of phenotype prediction models by LDA with LOOCV resampling. **Table S14.** relative expression level of candidate genes  measured by qPCR in populations ARA2, ARA8 and NAQ8 on plants in batches used for RNASeq (batches 1, 2 and 3a) and on an additional, independent batch (batch 4) (see Table S3). **Table S15.** Summary of the genes family associated with NTSR to herbicides in weeds identified in RNA sequencing studies ; genes identified in our study are highlighted in orange.

**Additional file 2: Supplementary Figure S1.** Geographical location of the six populations used in this study. The minimum distance observed between populations was 1.3 km (populations ARA2 and ARA8); the maximum distance was 395 km (populations NAQ8 and ARA2). This map is the authors' original artwork. **Supplementary Figure S2.** ALS gene expression measured by qPCR between all the resistant and all the sensitive plants of each population ARA2, ARA8, CVL5, NAQ8, NAQ9, OCC13 used in this study; p-value represents wilcoxon rank test results. **Supplementary Figure S3.** flow-chart of the experiments conducted. **Supplementary Figure S4.** Principal component analysis of the global expression profiles of plant RNA pools in batches 1 (top, before treatment (BT) modality, population ARA2) and 2 (bottom, before treatment (BT) modality, populations ARA8, NAQ8 and OCC13). **Supplementary Figure S5.** Principal component analysis of the global expression profiles of plant RNA pools in batch 3b (2 hours after treatment (2HAT) modality, populations ARA2, ARA8, CVL5, NAQ8, NAQ9 and OCC13).

## Data Availability

PacBio-RSII data and RNA-Seq data were deposited into the NCBI Sequence Read Archive database respectively under accession number SRP401902 and SRP401895. Reference transcriptome assembly sequence file is available under DOI: 10.25794/reference/3fb_9gip and a dedicated portal is publicly available at https://lipm-browsers.toulouse.inrae.fr/AMBELBase

## Abbreviations

ALS: Acetolactate-synthase
TSR: Target site resistance
NTSR: Non target site resistance
BT: Before treatment
2HAT: Two hours after treatment
RNASeq: RNA sequencing
DE: Differentially expressed
GO: Gene Ontology
LOOCV: Leave-One-Out Cross Validation
BP: Biological Process
MF: Molecular Function
