# Supplementary material for: A high diversity of non-target site resistance mechanisms to acetolactate-synthase (ALS) inhibiting herbicides has evolved within and among field populations of common ragweed (Ambrosia artemisiifolia L.)
Source: BMC Plant Biol. 2023 Oct 24;23:510. doi: 10.1186/s12870-023-04524-0 (PMC10594812; doi:10.1186/s12870-023-04524-0)
Supplement: Supplementary file 2 — Additional file 2: Supplementary Figure S1. Geographical location of the six populations used in this study. The minimum distance observed between populations was 1.3 km (populations ARA2 and ARA8); the maximum distance was 395 km (populations NAQ8 and ARA2). This map is the authors' original artwork. Supplementary Figure S2. ALS gene expression measured by qPCR between all the resistant and all the sensitive plants of each population ARA2, ARA8, CVL5, NAQ8, NAQ9, OCC13 used in this study; p-value represents wilcoxon rank test results. Supplementary Figure S3. flow-chart of the experiments conducted. Supplementary Figure S4. Principal component analysis of the global expression profiles of plant RNA pools in batches 1 (top, before treatment (BT) modality, population ARA2) and 2 (bottom, before treatment (BT) modality, populations ARA8, NAQ8 and OCC13). Supplementary Figure S5. Principal component analysis of the global expression profiles of plant RNA pools in batch 3b (2 hours after treatment (2HAT) modality, populations ARA2, ARA8, CVL5, NAQ8, NAQ9 and OCC13). [file 12870_2023_4524_MOESM2_ESM.docx]

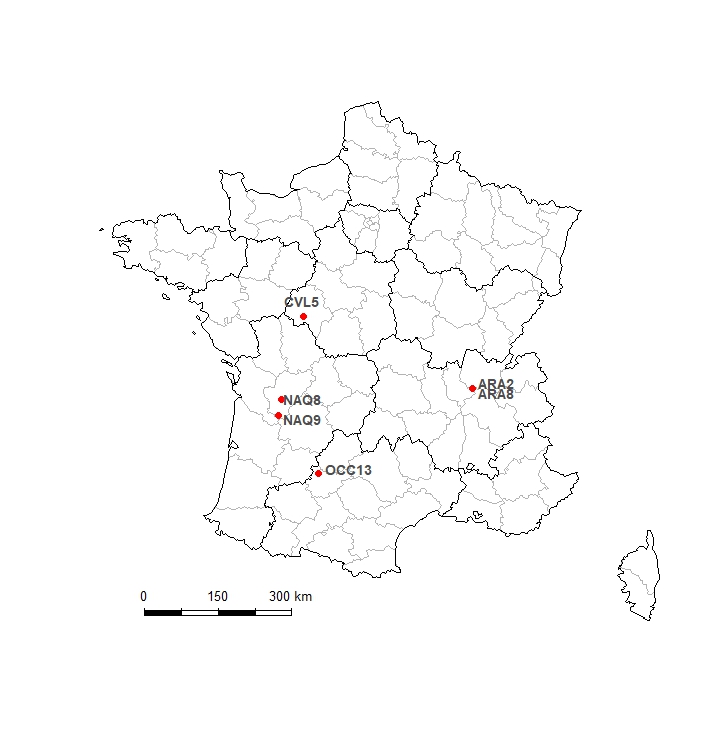


Supplementary Figure S1: geographical location of the six populations used in this study. The minimum distance observed between populations was 1.3 km (populations ARA2 and ARA8); the maximum distance was 395 km (populations NAQ8 and ARA2). This map is the authors' original artwork.


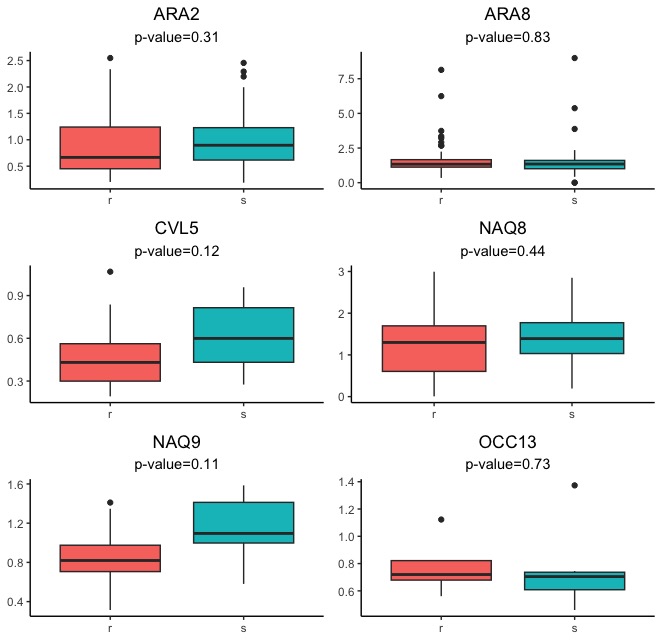


Supplementary Figure S2: ALS gene expression measured by qPCR between all the resistant and all the sensitive plants of each population ARA2, ARA8, CVL5, NAQ8, NAQ9, OCC13 used in this study; p-value represents wilcoxon rank test results.


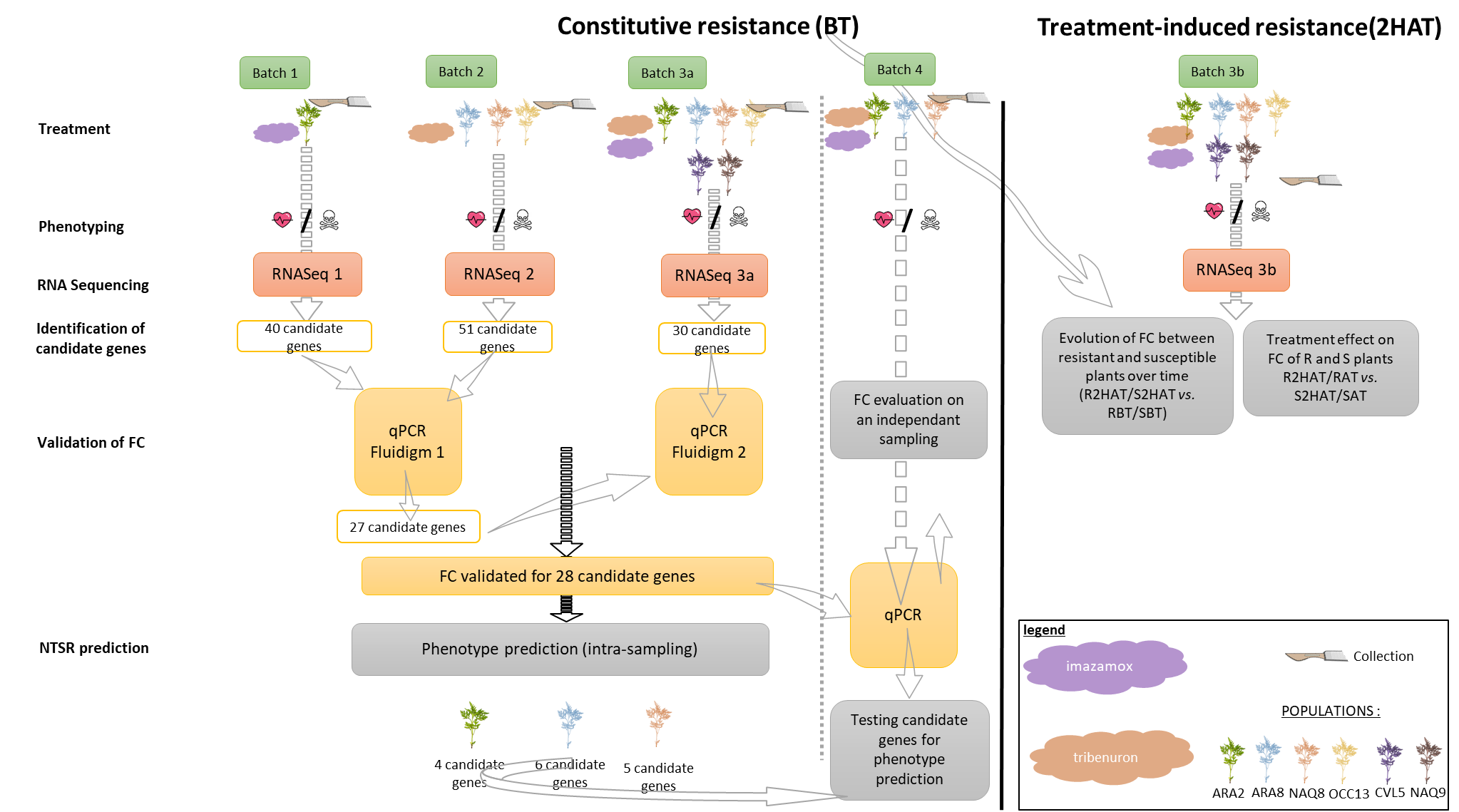


Supplementary Figure S3: flow-chart of the experiments conducted


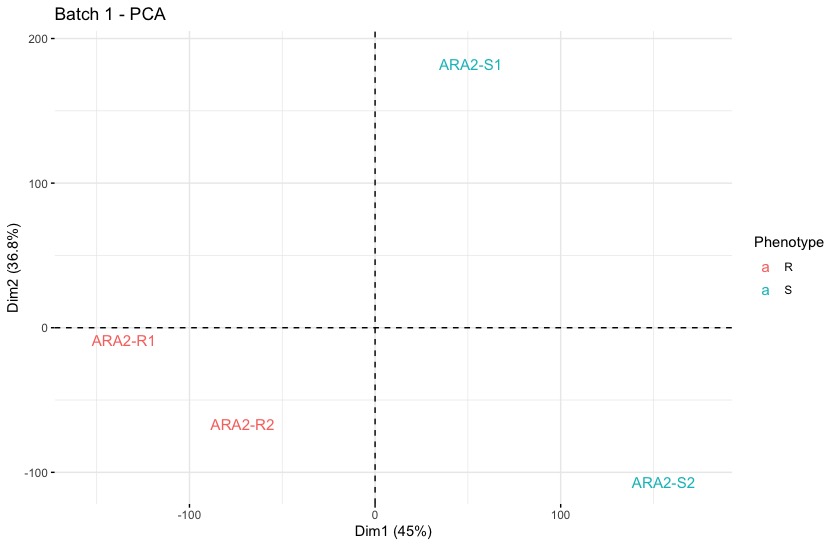


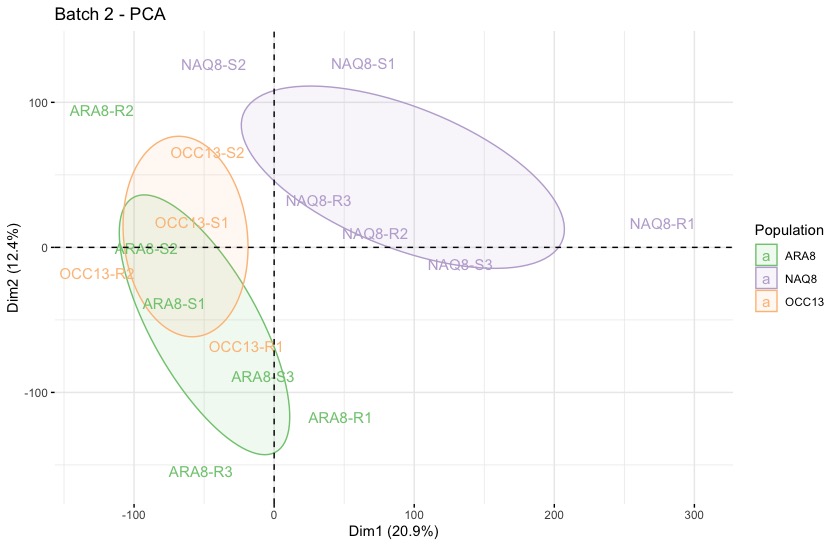


Supplementary Figure S4: Principal component analysis of the global expression profiles of plant RNA pools in batches 1 (top, before treatment (BT) modality, population ARA2) and 2 (bottom, before treatment (BT) modality, populations ARA8, NAQ8 and OCC13).


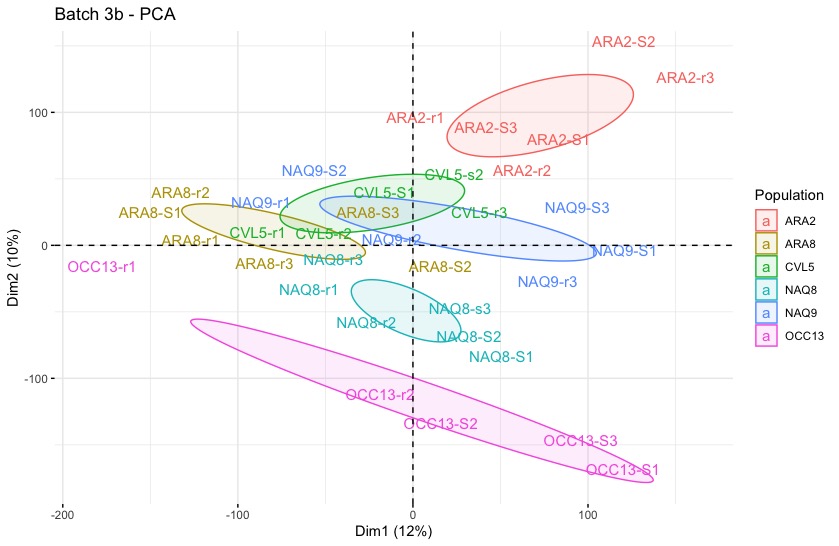


Supplementary Figure S5: Principal component analysis of the global expression profiles of plant RNA pools in batch 3b (2 hours after treatment (2HAT) modality, populations ARA2, ARA8, CVL5, NAQ8, NAQ9 and OCC13).
